# Comparisons of Quality of Life and Functional and Oncological Outcomes after Orthotopic Neobladder Reconstruction: Prostate-Sparing Cystectomy versus Conventional Radical Cystoprostatectomy

**DOI:** 10.1155/2017/1983428

**Published:** 2017-05-15

**Authors:** Po Yen Chen, Po Hui Chiang

**Affiliations:** Department of Urology, Kaohsiung Chang Gung Memorial Hospital and Chang Gung University College of Medicine, Kaohsiung, Taiwan

## Abstract

**Objectives:**

To compare health-related quality of life and oncological and functional outcomes on erectile function, continence, and voiding function among bladder patients who underwent orthotopic neobladder reconstruction after prostate-sparing cystectomy (PSC) and conventional radical cystoprostatectomy (CRC).

**Methods:**

This is a retrospective cohort study from a single surgeon. During 2007 to 2015, we identified 25 of 186 male patients receiving radical cystectomies due to bladder cancer who underwent orthotopic neobladder reconstruction, 14 patients with PSC and the other 11 patients with CRC. International Index of Erectile Function-5 (IIEF-5), International Prostate Symptom Score (IPSS), European Organization for Research and Treatment of Cancer-Quality of Life Questionnaire-Core 30-item questionnaire (EORTC-QLQ-C30), and self-catheterizations were used to evaluate functional outcomes in the baseline and 1 year after operation.

**Results:**

There were better physical and social functioning scales, less fatigue symptoms, better IIEF (16 versus 3.7, *p* = 0.01), and less self-catheterization rate (33% versus 89%  *p* = 0.006) in the PSC group. The oncologic outcomes were the same between two groups.

**Conclusions:**

For selected patients with bladder cancer who underwent neobladder reconstruction, prostate-sparing cystectomy provided better sexuality preservation, less daily self-catheterization, and better physical function and social function scales without compromising overall survival.

## 1. Introduction 

Conventional radical cystoprostatectomy (CRC) has been the standard treatment for patients with muscle-invasive bladder cancer or refractory superficial urothelial carcinoma. There are several surgical options of following urinary diversion from ileal conduit and various pouches to orthotopic neobladder reconstruction. Under the emphasis on functional outcomes and quality of life in recent years, the comparisons of health-related quality of life (HR-QoL) were reported among different diversions. In current evidence, orthotopic ileal neobladder demonstrates better HR-QoL in recent studies [1, 2]. For better functional outcomes, prostate-sparing cystectomy (PSC) with orthotopic neobladder reconstruction has been an alternative choice for selected patient groups without compromising oncological outcomes [3, 4]. Although many studies have reported better functional outcomes of PSC, few of the studies had a control group. Furthermore, there is a paucity of comparative studies regarding the HR-QoL difference in patients who have undergone CRC and PSC. Our aim is to make a two-armed study to compare the HR-QoL, functional outcomes, and oncological outcomes between patients who underwent CRC and PSC.

## 2. Methods

### 2.1. Study Design

The study proposal was approved by our Institutional Review Board (103-6330B) and this is a retrospective study of a single surgeon. We enrolled all the bladder cancer patients who received radical cystectomy in Kaohsiung Chang Gung Medical Center from 2007 to 2015. Only patients with at least one-year follow-up were included. Patients with non-cancer-related radical cystectomy or double cancer were excluded. To avoid bias of surgical technique, only cases performed by a single surgeon (Po Hui Chiang) were included. The selection of surgical type was suggested by the surgeon. If the bladder tumor was located near to the bladder neck, patients would be assigned to the CRC group. Suspicion of prostate cancer was also the contraindication of PSC. Otherwise, the patients would receive prostate-sparing cystectomy with informed consent. In PSC group, the prostate capsules, glands, and stroma were totally preserved; then Studer ileoneobladder was anastomosed to the outer rim of the prostate capsule after cystectomy. Routine frozen section of bilateral ureter and prostate urethra margin were sent. All patient data and questionnaires were collected by the same researcher to minimize performance bias.

### 2.2. Measurements and Statistics

We recorded age, BMI, Eastern Cooperative Oncology Group Performance Status (ECOG) before surgery, American Society of Anesthesiologists (ASA) Physical Status classification, pathological stage, neoadjuvant chemotherapy, a subject-completed European Organization for Research and Treatment of Cancer-Quality of Life Questionnaire-Core 30-item questionnaire (EORTC-QLQ-C30), International Index of Erectile Function-5 (IIEF-5), International Prostate Symptom Score (IPSS), day and night continence status, and self-catheterization times for all the patients before and at least one year after operation. A total of three measurements of IPSS score were recorded (before surgery, after surgery, and date of recent follow-up) for the evaluation of voiding function.

The EORTC-QLQ-C30 contains 30 items and incorporates a global health status scale, 5 functional scales, 3 symptom scales, and a number of single items assessing additional symptoms. We standardized the scores from 0 to 100. Higher scores on “Global Quality of Life” and “Functional Scale” represent a better quality of life, whereas higher scores on the “Symptoms Scales” reflect more intense complaints.

For statistics, we used SPSS 21 for data analysis, Chi-square test, or Fisher's exact test for association between two categorical variables. For small sample size, Mann—Whitney *U* test was used for comparison of medians of the age, BMI, and follow-up time between the patients in PSC and CRC groups. Kaplan-Meier survival estimates were obtained to compare the survival data and follow-up.

## 3. Results

### 3.1. Participants and Baseline before Surgery

There were 257 patients in total, with 186 males undergoing radical cystectomy in our hospital during 2007 to 2015 while 42 patients received orthotopic neobladder reconstruction. Among these patients, 6 patients underwent non-bladder cancer-related surgery and 9 patients underwent the surgery by other doctors. Besides, 2 patients were lost to follow-up from our department. Therefore, 25 patients were recorded in final statistics. The table lists the relevant patient characteristics (Table 1). There were no differences in age, BMI, pathological stage (T < T2 or T≧T2), pathological grade, IIEF-5, and IPSS scores between the two groups. All the patients before surgery had total continence and most of the men had intact erectile function (7 in CRC group, 10 in PSC group, resp.). No single patient had required self-catheterization in daily life before surgery. The EORTC-QLQ-C30 of the patients before the surgery also showed no significant differences in functional scales and symptom scales between the two groups (Table 2).

### 3.2. EORTC-QLQ-C30

HR-QoL assessment was performed according to EORTC-QLQ-C30 scales. Following surgery, after more than one year, there were no significant differences in global state of health between CRC and PSC groups. However, the physical functioning in CRC and PSC groups was 68.33 ± 27.54 and 95.15 ± 6.73, respectively (*p* = 0.029). Besides, better social function (68.75 versus 95.45, *p* = 0.017) and less fatigue (29.17 versus 8.08, *p* = 0.033) were also mentioned in the PSC group (Table 3).

### 3.3. Functional Outcomes

Our measurements of functional results included day and night continence, daily self-catheterization, urethral stricture, IIEF-5 score, and IPSS score. As for continence function, 4 patients still need pad at day time one year after surgery (2 patients in each group). The remaining 21 patients revealed no significant differences in day or night continence (Table 4). In analysis of self-catheterization, 8 out of 9 patients (89%) needed self-catheterization in their daily life in the CRC group compared to 4 out of 12 patients (33%) in the PSC group (*p* = 0.006). For patients needing CIC more than 3 times daily, PSC showed lower self-catheterization proportion (17%). Furthermore, there were 3 urethral stricture patients in the CRC group and none in the PSC group (*p* = 0.082). There were no significant differences of IPSS scores in both groups after surgery and 1 year after surgery.

In the measurement of IIEF-5 score, we excluded patients with moderate to severe erectile dysfunction (IIEF-5 less than 12) before surgery. Seventeen patients had intact erectile function before surgery. After surgery, IIEF-5 scores in CRC and PSC groups were 3.7 and 16.0, respectively (*p* = 0.014) (Table 4).

### 3.4. Oncological Outcomes

**Figure 1:**
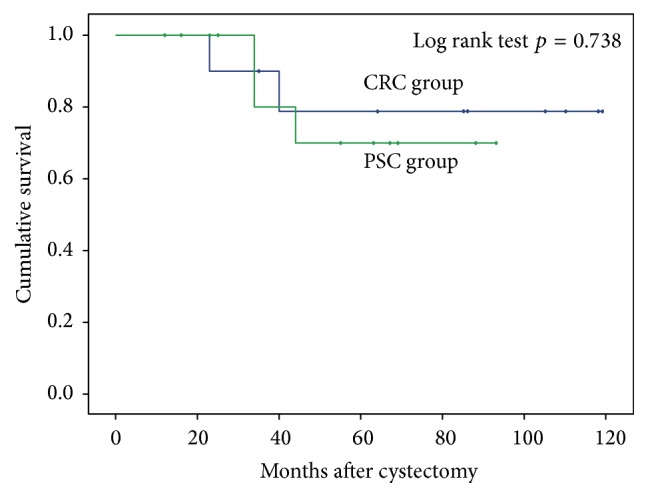
Kaplan-Meier survival curves in prostate-sparing cystectomy group and conventional radical cystoprostatectomy group.

In total, 27 men received radical cystectomy with neobladder reconstruction during 2007 and 2015; all patients except 2 patients lost to follow-up were followed in our outpatient clinics until 2016 after the surgery. Five patients (2 in the CRC group and 3 in the PSC group) expired during follow-up. Kaplan-Meier analysis for overall survival between the two groups showed no statistically significant differences between the two groups (Figure 1). Furthermore, 2 patients had distant brain metastasis (1 in each group). One patient in the PSC group revealed urothelial carcinoma following transurethral resection of the prostate due to urinary obstruction. Two specimens of radical cystoprostatectomy showed prostate invasion of urothelial carcinoma in the CRC group.

## 4. Discussion

### 4.1. Oncological Outcomes and Functional Outcomes

In a recent randomized controlled trial, prostate capsule-sparing and nerve-sparing radical cystectomy for bladder cancer reveal no differences of functional and oncologic outcomes [5]. However, total prostate-sparing including stroma still remains a controversial procedure for patients with muscle-invasive bladder cancer or refractory superficial urothelial carcinoma. Although it has been speculated that PSC preserves better sexual function and continence function, oncological outcome varies in different studies without consensus. Some studies disagreed with this operation due to inferior oncological outcomes [6–9]. Nevertheless, most of these studies did not reveal their own conventional radical cystoprostatectomy data compared to the PSC group. Furthermore, none of these had two-armed study designs. The only single matched-case control study showed better functional results without decreasing overall survival [10].

Some studies described invasive urothelial carcinoma or adenocarcinoma in the prostate specimens being found after radical cystoprostatectomy so that prostate-sparing surgery might not be performed in some circumstances [11, 12]. Yang et al. even reported up to 53% incidental prostatic adenocarcinoma or urothelial carcinoma invasions in Chinese patients undergoing radical cystoprostatectomy [13]. We did not perform preoperation prostate biopsy routinely in PSC group, but we have took the effort to rule out the patients with prostate cancer by digital rectal examination and PSA level. Although the proportion of patients with prostate cancer within normal PSA is very low, the prostate biopsy may still be a useful procedure in clinical practice for the patients planning for prostate-sparing cystectomy. However, if prostate cancer was detected during follow-up, we still have many further treatments such as radiotherapy, cryotherapy, HIFU, and even radical prostatectomy. As for urothelial carcinoma invasion, we sent frozen sections of distal urethra and prostate urethra for all the patients during operation instead of performing a transurethral resection of the prostatic urethra and prostate before cystectomy. In our study, two of 11 patients (18%) of the CRC group revealed urothelial carcinoma invasion and no incidental prostate cancer in the CRC group. Therefore, the preoperative transurethral resection of the prostatic urethra and prostate may be necessary in prostate-sparing patients to confirm that no prostatic urethral or stromal involvement exists.

This is not a randomized study. On the contrary, PSC was only performed in selected patients. All patients with tumors near to the bladder neck or suspicious of prostate cancer were excluded for PSC and it may illustrate this difference compared to previous studies.

In the largest prospective single-arm cohort study which involved 117 patients, better potency function without compromising oncological outcome was revealed [14]. Many reports have also shown no inferior cancer-specific outcome in the PSC group [3, 10, 15, 16]. In the present study, there were no significant differences of overall survival between the two groups. In terms of functional outcomes, the PSC group provided better erectile function and less daily self-catheterization.

### 4.2. EORTC-QLQ-C30

There were no compared studies discussing HR-QoL between CRC and PSC groups but only discussing factors influencing ileal orthotopic neobladder reconstruction. Imbimbo et al. mentioned that age, urinary incontinence, length of follow-up, and comorbidity status may influence postoperative HR-QoL in neobladder patients [17]. In our study, PSC surgery not only provided better physical function but also social function and less fatigue symptoms. Patients in the PSC group showed the trend of less night incontinence and significant difference of lower self-catheterization rates. This might be the reason for better health-related quality of life and even getting back to their work after surgery in younger patients.

## 5. Strengths and Limitations of the Study

To the best of our knowledge, our study is the first two-arm retrospective cohort study in Asia population which uses EORTC-QLQ-C30 to aim at HR-QoL and functional and oncologic outcomes in CRC and PSC patients with neobladder reconstruction in the same literature. Besides, only 7.4% loss of follow-up rate took place in our cohort study. However, there are still limitations in our study. Firstly, our patient allocation was subjectively dependent on a single surgeon's evaluation and tumor location. Although it revealed no statistical differences in terms of results of pathological staging and pathological grading between groups, selection bias might still exist. Secondly, the case number was small, so external validity power was limited, and, owing to lack of patient numbers, we could not perform subgroup analysis of oncological outcomes. Thirdly, patients might have had recall bias when taking the questionnaire.

We expect that large number control trials and even randomized control trials may be undertaken in the future. Further investigation is warranted to confirm the quality of life and the oncological results in prostate-sparing cystectomy with neobladder reconstruction. We concluded that prostate-sparing cystectomy can provide better functional outcomes and health-related quality of life without compromising survival in selected patients.

## Figures and Tables

**Table 1:** Patient characteristics.

Patient characteristics	CRC^†^ (*n* = 11)	PSC^†^ (*n* = 14)	*p*
Age	61.55 ± 15.03	57.5 ± 13.93	0.695
BMI^†^	23.85 ± 2.97	24.56 ± 4.71	0.695
ASA^†^	2.09	2.14	0.706
ECOG^†^	0.27	0.29	0.891
Neoadjuvant chemotherapy	1 (9%)	2 (14.3%)	1.000
Pathological stage:			
<T2 N0	6 (54.5%)	5 (35.7%)	0.437
T2 or higher or N positive	5 (45.5%)	9 (64.2%)	0.351
Pathological grade:			
Low grade or glandular cystitis	3 (27.2%)	2 (14.3%)	0.11
High grade	7 (64.6%)	12 (85.7%)	
Numbers of patients with intact erectile function before surgery	7 (64.6%)	10 (71.4%)	
IIEF-5^†^ for intact erectile patients	23.9	23.9	0.956
IPSS^†^	8.6	12.6	0.34
Daytime continence (%)	11 (100%)	14 (100%)	—
Nighttime continence (%)	11 (100%)	14 (100%)	—
Need CIC^†^	0%	0%	—
Follow-up time (months)	73.82	51.14	0.047^*∗*^
	(IQR^†^ = 84)	(IQR^†^ = 65)	

^†^ASA, American Society of Anesthesiologists Physical Status classification; ^†^ECOG, Eastern Cooperative Oncology Group Performance Status; ^†^CRC, conventional radical cystoprostatectomy; ^†^PSC, prostate-sparing cystectomy; ^†^BMI, body mass index; IIEF, International Index of Erectile Function; ^†^IPSS, International Prostate Symptom Score; ^†^CIC, Clean Intermittent Catheterization; ^†^IQR, Interquartile Range; ^*∗*^*p* < 0.05.

**Table 2:** EORTC-QLQ-C30 of the men before surgery.

EORTC-QLQ-C30^†^	CPC (mean ± SD)	PSC (mean ± SD)	*p*
Global health status/QoL^†^	61.11 ± 20.18	50.16 ± 23.14	0.569
Functional scales			
Physical functioning	75.55 ± 28.49	97.49 ± 3.45	0.118
Role functioning	83.33 ± 25.81	93.75 ± 17.67	0.419
Emotional functioning	84.72 ± 17.01	75.00 ± 23.14	0.404
Cognitive functioning	91.67 ± 9.13	95.83 ± 7.72	0.613
Social functioning	91.66 ± 9.13	95.83 ± 7.71	0.373
Symptom scales			
Fatigue	69.45 ± 26.70	11.11 ± 11.87	0.132
Nausea and vomit	0.00	16.66 ± 35.63	0.227
Pain	13.89 ± 19.48	16.66 ± 35.63	0.922
Dyspnea	0.00	8.33 ± 15.43	0.17
Insomnia	33.33 ± 42.16	12.50 ± 24.80	0.267
Appetite loss	0.00	12.49 ± 17.25	0.08
Constipation	20.83 ± 30.54	12.12 ± 22.47	0.773
Diarrhea	5.55 ± 13.60	4.16 ± 11.78	0.841
Financial difficulties	0.00 ± 0.00	0.00 ± 0.00	—

^†^EORTC-QLQ-C30, European Organization for Research and Treatment of Cancer- Quality of Life Questionnaire-Core 30-item questionnaire; ^†^QoL, quality of life.

**Table 3:** QLQ-C30 of the men after surgery at one-year follow-up.

QLQ-C30	CRC (mean ± SD)	PSC (mean ± SD)	*p*
Global health status/QoL	34.38 ± 18.05	29.55 ± 23.08	0.63
Functional scales			
Physical functioning	68.33 ± 27.54	95.15 ± 6.73	0.029^*∗*^
Role functioning	83.33 ± 21.82	96.97 ± 10.04	0.134
Emotional functioning	95.83 ± 8.91	96.97 ± 10.05	0.802
Cognitive functioning	89.58 ± 12.40	92.42 ± 11.46	0.613
Social functioning	68.75 ± 24.30	95.45 ± 10.78	0.017^*∗*^
Symptom scales			
Fatigue	29.17 ± 22.17	8.08 ± 10.05	0.033^*∗*^
Nausea and vomit	8.33 ± 23.57	24.24 ± 30.15	0.232
Pain	10.42 ± 17.68	1.52 ± 5.03	0.204
Dyspnea	12.350 ± 24.80	15.15 ± 22.92	0.813
Insomnia	33.33 ± 35.64	12.12 ± 16.82	0.1
Appetite loss	4.17 ± 11.78	15.15 ± 31.14	0.359
Constipation	20.83 ± 30.54	12.12 ± 22.47	0.482
Diarrhea	4.17 ± 11.78	3.03 ± 10.05	0.824
Financial difficulties	0.00 ± 0.00	0.00 ± 0.00	—

^*∗*^ *p* < 0.05.

**Table 4:** Functional outcomes after one year of surgery.

	CRC (*n* = 11)	PSC (*n* = 14)	*p*
Day continence	9 (82%)	12 (86%)	0.754
Night continence	6 (55%)	9 (64%)	0.746
CIC	8 (89%)	4 (33%)	0.006^*∗*^
Daily CIC > 3 times	6 (67%)	2 (17%)	0.018^*∗*^
IPSS after surgery^†^	16.6	12.2	0.302
IPSS 1 year later^†^	17.5	7.5	0.059
Urethral stricture	3 (27%)	0 (0%)	0.082
IIEF-5^‡^	3.7	16	0.014^*∗*^
Morning erection	1 (14%)	7 (70%)	0.022^*∗*^

^†^Total incontinent patients (2 patients in both groups) were not in the IPSS and CIC analysis; ^‡^IIEF-5 analysis for patent erectile patients: 7 patients in CRC group, 10 patients in PSC group; ^*∗*^*p* < 0.05.
